# Talent has no race, has no face - but it has a skin colour: The ‘appropriate femininity’ with the case of Kurdish-Swedish Actress Evin Ahmad

**DOI:** 10.12688/openreseurope.16156.1

**Published:** 2023-11-10

**Authors:** Özlem Belçim Galip

**Affiliations:** 1 University of Oxford, Oxford, England, UK

**Keywords:** Swedish film industry, appropriate femininity, feminist film theory, controlled images, whiteness, stereotype

## Abstract

Sexist and misogynist attitudes towards actresses in the mainstream film industry and other media have been the target of feminists for decades. Stigmatized and stereotyped images of immigrants on the screen have also been scrutinized. However, very little attention has been given to the ways in which actresses with foreign background, not necessarily from Black communities, are portrayed and narrated. Addressing this issue would reveal how non-Western body images on screen are racialized according to certain Western beauty standards.

Sweden is often described as the most gender-equal film industry in the world; however, this does not mean that marginalization and subordination of non-white actresses with foreign backgrounds, does not occur. Accordingly, using the framework of feminist film critique and drawing on the works of Homi Bhabba, Sara Ahmed and Patricia Hill-Collins, this article offers a reading of Swedish-Kurdish actress Evin Ahmad’s experiences to demonstrate how ‘beauty’ and ‘body’ culture involve a complicated terrain around race, skin colour and body image. This article argues that a generalized ideology of beauty and stereotypical images of a Middle Eastern woman imposed on Ahmad demonstrate that values and attributes such as beauty, appearance and sexual attractiveness should be understood in the context of social and cultural relations, rather than as universally valued or devalued individual characteristics.

## Introduction

Since 1970s, feminist film critics such as Mary Ann Doane, B. Ruby Rich, Laura Mulvey, Barbara Creed and Teresa de Lauretis have applied an intersectional lens to the issues of subjectivity, forms of female agency and cinematic representation, as well as the position of women in the film industry, from actresses to technicians. Black feminist critics like bell hooks and Michele Wallace have opposed the ‘totalizing agenda’ of feminist film criticism because it excludes multiracial and multi-ethnic aspects, though earlier theoretical intervention by Black feminists such as Audre Lodre, Barbara Smith and June Jordan had brought the question of the intersection of ‘gender’ with ‘race’ and ‘class’ to feminist debates. Since the black/white binary defines the history of US racial discourses, the focus has been on the essentialized roles or lack of leading roles played by black women, but this has expanded recently to include Hispanic and Latino or Asian women in the American film industry. ^1^ Drawing on feminist film theory from a marginalized perspective, the centrality of the body on screen ( Coleman, 2008; Doane, 1980; Kyröla, 2016) has been examined, and how African/Black women’s bodies are objectified in both everyday life and media, in contrast with the perceived beauty and purity of women of European origin ( Adelman & Ruggi, 2008; Hill-Collins, 2000; Mask, 2009).

However, in the context of Europe, the empirical research in media and film studies hitherto focuses mainly on the representation of migrants and descendants of non-Europeans/non-natives in contemporary European cinema ( Çelik Rappas & Phillis, 2018; Loshitzky, 2010) or migrant and diasporic film making ( Ballesteros, 2015; Berghahn & Sternberg, 2010; Ponzanesi, 2012). In this sense, however, the experiences of non-white or non-European actresses (not necessarily from Black communities) in European film industries are understudied. Some reports and interviews refer to the fact that non-European actresses are given stigmatized and essentialized roles which lack an intersectional dimension. ^2^ However, there is no in-depth analysis of the experiences of non-native actresses involved in the European film or TV industry, especially in Nordic countries, where, according to the statistics, there is a higher level of gender equality. This article examines how actresses with foreign background are received by the film industry in Sweden, which has an international reputation for its generous immigration policies for the inclusion of migrants ( Castles, 1995; Dahlstedt *et al.*, 2017).

Swedish-Kurdish actress Evin Ahmad, born in 1990 in Stockholm, has been selected as the case study for this article. European Film Promotion (EFP) announced the ten emerging European talents for 2022 for the 25th anniversary edition of *European Shooting Star* and Evin Ahmad was chosen as one of them ( European Film Promotion, 2022). She has recently become one of Sweden’s most sought-after talents, appearing in several feature films and a Swedish/Danish produced Netflix series, making her a good case study as we attempt to understand how her non-Swedish ethnic background has affected her successful career and her personal experiences, on and behind the screen ( Kapoor, 2023).

When meeting Evin in 2019, I intended to understand her lived experiences as a second-generation migrant and a well-educated young woman in a creative industry ( CORDIS, 2023). ^3^ However, her statements on the perception of her ‘skin colour’ on the screen diverted me towards thinking about how female sexuality and the standards of beauty and aesthetics of non-native women converge in the issue of race and gender on Swedish screens. This article does not aim to question whether she could obtain lead roles (as it is assumed that would be the case for actors or actresses with foreign background) or be acknowledged for her acting. She has clearly received recognition and acclaim for her work and taken a number of lead roles. Instead, the questions are: Has her non-white, Middle Eastern race resulted in essentialist narratives and racialized roles driven by Eurocentric values? ^4^ How does her skin and ethnic background manifest the social and discursive construction of her gender and race on the screen? The answers to these questions are sought through qualitative method involving a number of in-depth in-person interviews with Evin and by closely following her acting career through non-participant observation between 2019 and now. ^5^ Meeting her through the years has enabled me to observe the changes in her role selections and her thinking on the film/media industry in the Nordic and more general Western, context. Firstly, this essay will provide a brief introduction to the current state of play regarding gender equality in the Swedish film industry, along with an analysis of Swedish immigrant integration policies and their impact on the Kurdish diaspora.

## Gender equality in the Swedish film industry: Is it good enough?

Although it has been widely argued that the gender equality measures undertaken in Sweden are insufficient to come to grips with the gender inequalities in the film industry, looking at Europe, the weighted average of films directed by women in the 2012–2016 period is just 19.6%, with country results varying from 5% (Latvia) to 30% (Sweden) ( Katsarova, 2021), showing the success of Sweden compared to other European countries. However, this success has taken a long time. Despite the efforts of women film workers the 1970s, it was only in 2000 that the Swedish government formally tasked the Swedish Film Institute (SFI) with working towards greater gender equality and with monitoring the progress of female film projects funded through the Institute ( Jansson & Wallenberg, 2020). One of the SFI’s goals is to lay the foundation for gender equality in film production, a labour that it is able to progress with the aid of concrete action plans, beginning in July 2016 with “Goal 2020: Gender equality in film production, both in front of and behind the camera” ( Swedish Film Institute, 2021). In the same year it launched a “FiftyFifty by 2020” event at the Cannes Festival to promote Sweden’s stance on gender equality in film production and to raise international awareness on the issue. In 2020, overall the same proportion of characters were female as were male which is a big achievement compared to many Western countries including the USA ( Wallenberg, 2023). In 2019, females accounted for 37% of major characters and 68% of all female characters with speaking roles were White, 20% were Black, 5% were Latina, 7% were Asian, and 1% were of some other race or ethnicity ( Lauzen, 2019). More women are involved in the film industry in Sweden, with an equal share of women directors, producers and scriptwriters ( Byrnes, 2015; Jansson *et al.*, 2021). However, this does not mean that women are still not disadvantaged in terms of budget size or distribution or that there has been no discussion of overt sexism in the industry. Hence, it is argued that the gender equality measures undertaken in Sweden are not sufficient to come to grips with the gender inequalities in the industry ( Jansson & Wallenberg, 2020).

Gender inequalities and the stereotypical representation of women on stage, in theatres and in TV also constitute a real battlefield for the new generation of female artists in general ( Wing-Fai *et al.*, 2015). According to Jansson and Wallenberg (2020) it is the financiers, producers and distributors rather than the directors who request stereotypical portrayals of female characters in the Swedish film industry. The *Which Women?* Gender Equality Report 2019–2020, a qualitative research study on the working conditions for older women and women who are being racialized in the Swedish film and TV industry, indicates that the industry is at risk of losing relevant talent and missing out on important stories by continuing to reproduce stereotypical social imagery. ^6^ The Swedish Film Institute therefore concludes that diversity and increased representation pose untapped potential in Swedish film, both commercially and qualitatively. Again, the report indicates that there is a distinct fixation on youth and certain beauty ideals in the industry, leading to an underrepresentation of groups in society that do not meet those standards. Although this report does touch upon hegemonic portrayals of women with certain beauty features, it does not specifically reflect the experiences of the descendants of non-Swedish actresses when they are subject to these beauty standards or examine how these standards intersect with their racial and ethnic background.

## Evin Ahmad: A positive outcome of Sweden’s immigration integration model

Sweden has one of the largest proportions of immigrants in relation to the total national population in Europe. In 2020, every fifth inhabitant of Sweden was born outside the country, and over a quarter had a migrant background ( Hajighasemi & Oghazi, 2021). In the context of post second world war policies on migration after the reception of large groups of refugees and then labour migrants from the late 1950s to the official halt to immigration in 1972, Sweden started to develop a specific policy approach to promoting immigrants’ integration into Swedish society influenced by universal welfare state principles and the Social Democratic welfare state regime ( Borevi, 2012). Although from 1972 onwards, the refugees were mainly replaced by labour migrants from non-European countries, the Kurdish influx, especially between the 1980s and early 2000s, mainly consisted of asylum seekers due to the intensified political conditions in their home countries, Iraq, Iran, Syria and Turkey. Kurds, the biggest stateless nation in the world with an approximate population of 40 million, are divided between these four countries but, especially in the last four decades, are also dispersed all over the world for socio-economic and political reasons,.

Sweden has been considered one of the most migrant-friendly European Union (EU) member states and one of the most diverse European societies ( Schierup & Ålund, 2011). ^7^ Kurds followed their familial and political networks, which led to the concentration of the Kurdish Diaspora in certain countries, with Sweden being among the leading ones alongside Germany, France and the Netherlands. ^8^ Access to equal rights such as welfare state services and benefits, social equality, was regarded as a necessary condition for their integration into Swedish society ( Borevi, 2012) together with affirming and supporting their ethnic identities. Like other immigrants and their children, they were given the possibility of retaining their own language, practicing their cultural activities and maintaining contact with the home country. Kurds have benefited from reforms including support for journals produced in Kurdish, Kurdish as mother tongue instruction in the public school system and financial support for ethnic or community organizations. Overall, one can confidently state that these reforms have enabled the Kurds to maintain their ethnic affiliation and be integrated to Swedish majority culture. ^9^ Although the model of multiculturalism has been critically debated, Sweden’s integration and immigration policies have created a positive environment for Kurds where they could be both socially, politically and culturally active ( Eliassi, 2016; Khayati, 2008). ^10^ In this context, Kurds from all four Kurdish regions have been active in the Swedish parliament and have been involved in film production, radio broadcasts and exhibitions, as well as in the production of a substantial number of books, journals and other publications in both Kurdish and Swedish. The social and economic crisis of the early 1990s meant that economic constraints causing austerity measures made it harder to finance generous social policies on mother-tongue publications or financing community centres. The European refugee crisis of 2015 led to path-breaking policy changes in a very restrictive direction in Sweden, with tough implications for new arrivals or those wishing to arrive, while second generation Swedish Kurds, having already acquired a Swedish education and awareness of the system, appear in various creative sectors with their hybrid identities.

Cinema, media and theatre are creative fields alongside literature and music in which Kurds have been in evidence. Undoubtedly, actress Evin Ahmad, born in 1990 in Stockholm, is one of the most well-known. According to the Jury of European Film Promotion (EFP) for European talents (2022),

Evin Ahmad radiates unique energy which is both enigmatic and approachable. Smoothly integrating many sides of the same coin, she exudes strength, resilience, vulnerability, as well as a self-aware sense of humour. Her charm is natural and unpretentious, while her glamor and playfulness contribute to her potentially dangerous beauty. We see a very bright future ahead and foresee a lot of international opportunities.

Having been nominated twice for best actress in supporting roles at the Guldbagge Awards (the official annual Swedish film awards) and being selected as the Shooting Star of Sweden in 2022, inability to obtain a leading role has never been an issue for Evin (in contrast to common thinking about non-white actresses). Despite white privilege or racial segregation in the film industry, her talent and determination have resulted in Evin playing several leading roles in Swedish in feature films including *Beyond Dreams* (2017) and *Ring Mamma* (2019), TV series such as *Tsunami* (2020) *, Max Anger-With one eye Open* (2021 *), Snabba Cash* (2021–22) and Danish sci-fi drama Netflix series *The Rain* (2018–2020). ^11^ She also took the leading role alongside Alexander Salzberger at The Swedish Royal Dramatic Theatre in the play *Determinism* (2020), written by well-known director and scriptwriter Mattias Andersson. ^12^ It should be acknowledged that Evin creates her chances by pursuing her dreams of what she really wants to achieve in her acting career. She even emailed the director of *Determinism* when she was still studying drama and told Andersson that she wanted to work with him in the future, which eventually happened almost a decade later.

Evin started her acting career when she was 15 in the role of a troubled migrant teenager. As a descendant of refugee Kurdish parents, living in working class suburbs, she says that becoming an actress is beyond the dreams of the neighbourhood she grew up in. ^13^ It has been shown that those of a first- and second-generation non-Western migrant background experience lower employment chances, especially in the creative sector, with more skill mismatches and lower job satisfaction compared to natives of the host countries ( Belfi *et al.*, 2022; Zwysen & Longhi, 2018). This echoes feminist scholar Sara Ahmed’s personal remark on how her Muslim name slows her down, “to inherit a Muslim name in the West, is to inherit the impossibility of a body that can ‘trail behind’, or even to inherit the impossibility of extending the body’s reach” ( Ahmed, 2007). This relates very much to how career plans in creative or artistic fields are considered by members of ethnic communities, notably parents, to be risky, unstable, unachievable and unrealistic. Accordingly, Evin Ahmad says:

When I was a kid, nobody told me that you need to become an actress, or you have so much talent. Or you should be writer. They were just telling maybe you should be hairdresser… or you could be… a nurse. ^14^

However, Evin’s family supported her, and at the suggestion of her teacher at high school, she got the role of Magda after being auditioned when she was a teenager. She is grateful that her debut role in *Till slut* (In the End, 2007), directed by Maria Hedman, based on a novel by Mare Kandre, allowed her to pursue her true passion of acting professionally, leading her to study acting at Stockholm Academy of Dramatic Arts. However, she is also critical of the fact that her first role, as troublemaker second-generation migrant, was representative of a stereotyped image of migrant communities in Europe. She points out that education, self-improvement and intellectual thinking brought her to a stage where she knew she should stay away from roles which employed stereotypes. This was especially the case as she is a Kurd, and therefore linked to honour killings, as Fadime Şahindal’s (1975–2002) case affected the whole of Kurdish-Swedish society in a very deep and detrimental way ( Wikan, 2008). Fadime was murdered by her father in Uppsala in January 2002 in the name of honour killing. What Şahindal’s father did was recorded as having been done in order to protect Kurdish traditions. Media organizations and the authorities all interpreted her father’s oppressive attitudes towards her daughter as being a consequence of Kurdish traditions rather than regarding him as an individual obsessively controlling father ( Mojab & Hassanpour, 2002). This was not questioned much, and the identification of Şahindal’s murder in the name of honour with Kurdish society still resonates even after almost 20 years. Evin deliberately refuses roles involving women linked to honour killings in order to subvert the identification of Kurds with such honour related crimes. This relates to Hazel Carby’s (1987) suggestion that the objective of stereotypes is “not to reflect or represent a reality but to function as a disguise, or mystification, of objective social relations.” In relation the institutionalized prejudices, racial stereotypes, and representation that’s indirectly imposed on her, Evin says, “I am expected to wear hijab on the screen, as an Asian woman is expected to be a prostitute. In the line of colonialist rules, I can never simply be a beautiful and romantic girl just living next door”. ^15^ In fact, the idea of ‘ *representation’* is often used in the application of critical race theory, describing how a single image of an ethnic character can manipulate the way we form stereotypes and how we consider that ethnic group as a whole for the sake of ourselves ( Armstrong, 1996). Accordingly, Walter Lippmann, who coined the term ‘stereotype’, says stereotypes are “the fortress of our tradition, and behind its defences we can continue to feel ourselves safe in the position we occupy” ( 1956).

Coming from a Black feminist perspective, American scholar bell hooks (1992) promoted the notion of the ‘oppositional gaze’, encouraging black women not to accept stereotypical representations in film, but rather actively critique them. Correspondingly, Evin has not only strived to reject stereotyped roles but she has become critical about it to the extent that she has carved a tattoo of the term ‘stereotype’ on her wrist, as a counternarrative against stereotyping. She says:

You do not want to be stereotyped. My first acting experience the role of a stereotyped troubled migrant teenager. I did not have any experience back then, so I jumped onto it. But when I look at my career at this moment, I started 15 years ago. I got the formal education, I got better and better, developed my techniques, worked very hard and had a lot of experience in doing different roles; people know me now. They would not just label me with certain roles based on my skin colour […] It is true that talent has no race, has no face; however we live in Europe, and it is very segregated. ^16^

## ‘Controlling images’: objectification of ‘other’ on the screen

Linked to the construction of the ‘other’ in Edward Said’s classic work on Orientalism and postcolonial feminist scholarship, in which non-western women are portrayed as exoticized and defended as victims of uncivilized cultural practices, this is very much the case of ‘other’ on the screen. The Anglo-American film industry has produced many distorted representations positioning screen characters as ‘other’ because of their designation as nonnormative (whether black, gay, migrant etc.). In this regard, Evin says that from the start of her career she has been cautious about mystified or exotified roles which promote an image of the subordinated or victimized Middle Eastern or migrant woman. Patricia Hill Collins’ term ‘controlling images’ refers to stereotyping of Black women ( 1998; 2000), but can be applied to non-white or ethnic groups. The news media and government agencies constitute important sites for reproducing such images of predominantly victimized migrants or refugees. The use of ‘controlling images’ in the film industry is very much in parallel with the creation and dissemination of dominant media portrayals of migrants and refugees. The refugees on boats attempting to cross the channels from third world countries are, in a way, imposed on Evin, and the ‘victim’ image can be delineated through certain behaviours (obedient, fearful etc.) and oversimplified portrayals of Middle Eastern characters, leading to ‘objectification’. Anthropologist and African Studies scholar Dona Richards (1980) states that the West needs ‘objectification’ as it separates the ‘knowing self’ from the ‘known object’ in order to maintain white privilege. One type of objectification Evin encounters in her profession is simply being slotted into the homogenous and essentialized role of ‘Middle Eastern woman’, lacking any distinctive identity codes. In relation to such ‘controlled’ roles, she states:

Most of my characters if I list them, are always named Sara. Because it is sort of a neutral name. Or they want to name me Fatima or Hasmin. OK, that is fine, but then where is she from then?[…] Is she just Middle Eastern?... You know there are a few countries in the Middle East… There are, for example, Kurdish people, so why are you not specific…?

Apart from the victimized and stigmatized migrant/refugee image, another powerful ‘controlling image’ applied to those arriving from the Middle East arises from the fact that they are highly politicized and very much involved with politics in their daily lives due to the unstable political environment in the region. Therefore being of Middle Eastern origin, even though second generation, born in Sweden, Evin is expected to be well informed about any political changes in the Middle East, an expectation which comes at the expense of attention being focused on her as an actress. Hence, her racial identity invokes historical constructs, but Evin resists being objectified as ‘other’ in the form of a piece of documentary for the spectators. Objectification can be so severe that the ‘other’ simply disappears, says Black feminist theorist Collins (1998). In order not to be objectified, she resists allowing her racial background to get in the way of her acting career or overshadowing her professional passions. She complains that when she meets Western journalists to talk about her films or acting career, they constantly ask questions about ISIS (the Islamic State of Iraq and Syria). “When they look at me, they see a documentary not an individual”, she states. Her Syrian Kurdish background, attracting more attention than her acting, not only overshadows her career, but it also leads to a stigmatized and marginalized portrayal by film critics and journalists. Evin tells me of one such experience:

One day I did a big interview for a big magazine. The journalist came to the theatre and I showed all my theatre and film posters with my face on them. I had done mostly leading roles at that time. I talked about my philosophy and my life in the interview. But she put big headlines in her article saying ‘Evin is also dreaming about the big roles’. I called her afterwards and asked her, ‘did you miss that my face was all over? Why do you want to victimize me?’ She said ‘it is not interesting to talk about an actress who has got all. I wanted to write about a girl who is very moving and emotional’. I said ‘you cannot do this. If you want to do an emotional or a moving story, go and write about war or sexual assault or something like that. I am here as an actress. I am here to be on exactly the same terms as my colleagues. ^17^

This is a good example of how in media discourse in a receiving country context, migrant child or non-white identifiers are linked to certain inherent values (e.g. unfulfilled dreams, disappointment), which do not change even when the reality clearly shows the opposite. “Regardless of any individual woman’s subjective reality, this is the system of ideas that she encounters. Because controlling images are hegemonic and taken for granted, they become virtually impossible to escape,” says Collins (1998: 90). Accordingly, to bell hooks, while subjects have the right to define their own reality, establish their own identities, name their history, as objects, one’s reality is defined by others, one’s identity created by others, one’s history named only in ways that define one’s relationship to those who are subjects ( 1989: 42). In this sense, Evin’s reality has been defined by her racial identity as created by others. However, as cinema is believed to represent and narrate ‘race’ ( Bernardi, 1996), Evin is adamant in refusing to solely represent her ‘race’ in regard to acting on stage or screen. This explains why she wholeheartedly accepted the role of Marie Antoinette in a play at a regional theatre (Folkteatren) in Gothenburg in 2017, one of various productions where she has played upper class roles. The central polarity between white Europeans and non-white others lies in the way physical attributes are understood as signifiers of people to whom differing social and historical processes are seen to apply. Although she received positive reviews for her acting, the fact that the French queen was played by a non-white also created “undefined discomfort”, says Evin. She asserts: “Because I am a Kurdish in origin, I was not expected to play a white queen from the 17 ^th^ century. Here I am. I show you that I am an actress, it is my job to do other people. I do not have to be a documentary; I don’t always have to portray a Kurdish girl scared of being hit by her father”. Cinema, as in feminist film theorist Laura Mulvey’s words, has become the crucial terrain on which feminist debates about culture, representation and identity have been fought (1989). Accordingly, Judith Butler claims that presumption of a binary gender system implicitly retains the belief in a mimetic relation of gender to sex whereby gender mirrors sex. Nowhere has this mimetic relationship between sex and signifiers of gender been more apparent and more regularly inscribed than in the cinema. Bodies are not given as nature but inscribed within particular cultural formations ( Grosz, 1994). From the lens of phenomenology, Ahmed’s approach ( 2004; 2007) to ‘whiteness’ related to how it orientates bodies in specific directions, affecting how they take up space and what they can do is useful in helping us to understand how ‘whiteness’ becomes a background to social action. Within these terms, ‘the body’ becomes a passive medium on which cultural meanings are inscribed; in this case someone who is not a complete ‘white’ cannot play a ‘white’ role, in turn reproducing ethnocentrism in the film industry.

## #She too? Tedious ‘non-whiteness’ versus pretty white ‘exclusivity’

‘Whiteness’ as an emergent subject of academic inquiry has crossed a number of disciplinary boundaries, to be informed by the work of sociologists, anthropologists, literary scholars, and historians. ^18^ In fact, the influence of whiteness as a yardstick for beauty has a history which extends back to slavery ( Bush, 1990; Tate, 2007) and the desire for lighter skin continued into the colonial period ( Fanon, 1967). The white French queen is not the only ‘body’ of which Evin’s personification has given rise to doubt, there are others that draw on experiences of inhabiting a white world as a non-white body, showing how whiteness becomes worldly through the noticeability of the arrival of some bodies more than others. Sexual harassment is seemingly just the tip of the iceberg in an industry where gender inequalities relating to biased representation and pay are, it seems, systematic and pervasive. I would not have been surprised to hear Evin giving examples of sexual harassment in the context of the ‘#metoo’ movement, especially following the sexual abuse allegations against the American film producer Harvey Weinstein, which led to the silence being broken and many hitherto supressed stories of sexual misconduct in the film industry coming to light ( Gorman, 2023). However, Evin’s take on the ‘#metoo’ campaign was very different from those women who had revealed their experiences of sexual assault or harassment in the film industry in Europe and in the USA. Evin was aggrieved by the way her roles were ‘desexualized’ as a consequence of her ethnic background, being non-white and therefore, according to her, being treated as a less sexualized being. She says:

When all the actresses all over the world stood up against sexism in the industry, in the beginning, I could identify myself with sexism because I am a woman, but in this industry in general, not in Sweden. My body is not sexualized. In the scenes, my body gets desexualized. The characters that I play rarely fall in love or have sex or are involved in any kind of sex scenes. ^19^

After doing a little research and analysis on the roles played by non-Swedish, non-white, non-blonde actresses, she saw the same pattern of ‘desexualization’. She continues:

When I look at all the beautiful Indian, Black, Middle Eastern actresses in Sweden, I see that none of them are considered as physically attractive or appealing by film or TV standards. I come to the set, the production team says, ‘you are so beautiful’, but you sit in a make-up room and wait for somebody to do your make-up but then they say, ‘your character does not wear make-up’. The clothes picked for us are not even proper dresses. Just very ugly outfits not even a dress. In fact, our body can turn into any character that we portray. We have a complex body. We can be sexy, we can be romantic, we can be angry, and we can be frightened. But whenever I do a character, they are always one thing. They are not shot in either erotic or romantic scenes. ^20^

The fact that Evin is considered less feminine by the film-making team because she occupies a marginal subject position demonstrates that the associations between gender, sex and desire are themselves predicated on the hyperrational, patriarchal social order of Western societies ( Mask, 2009). Notions of femininity and feminine beauty are still predicated on bodily signifiers in Euro-American cultures as part of a privileged historical discourse on race which is dominated by the aesthetic project of presenting ‘white’ as an ideal, the standard by which to measure beauty. In this regard, Evin says: “If I was white, they would find me attractive. I have never been considered as attractive. That’s also racism and sexism.” ^21^ Here she encounters racism as an aesthetic project, one that seeks to define and appropriate what is beautiful while defining ‘others’ as ugly. Thus, prevailing standards of ‘beauty’ mean that no matter how intelligent, educated or ‘beautiful’ a non-native is, within the binary thinking that underpins intersecting oppressions, blue-eyed, blonde, thin and white women are assumed to be the ideal, and those with white skin are privileged in a system that disparages non-whiteness.

In contrast to Sharon Willis’ (1997: 1) observation on popular representations which have “amplified these eroticizing impulses by elaborating social differences as aesthetic or sensational effects”, in Evin’s case the reproduction of aesthetic standards happens through ‘desexualization’ in which she is ‘defeminized’ or ‘desexed’ as the embodiment of unattractiveness. In the hegemonic masculinist norms of the film and TV industry, Evin is expected to deny her female sexuality, portraying submissive, dependent roles which again recall Collins’ ‘controlled images’. Evin is turned into a controllable form exhibiting constrained forms of bodily pleasure and expression as she does not tick the box of ‘appropriate femininity’. ‘Appropriate femininity’ here means being ‘white’.

Feminist theory points out that patriarchal culture embodies women as ‘objects of the male gaze’ as part of hegemonic commercial culture. Hence, cinematic pleasures operate for the male spectator, whilst the figure of ‘woman’ functions as fetishized object of desire or object of narrative punishment ( Mulvey, 1975). However, although Evin meets the socially accepted standards of ‘beauty’ in the context of thinness, youth, heterosexuality, and ability ( Bordo, 1993; Bordo, 2003; Tiggemann & Lewis, 2004), she is still not considered beautiful or sexual enough to be given relevant roles. In this case, the reproduction of defective or disliked femininity for non-white actresses in the film industry in Sweden functions to sustain the institutional dominance of ‘appropriate femininity’ for the sake of the white ‘male gaze’.

In this context, being a white actress on the screen results in one becoming the legitimate object of the ‘male gaze’. In other words, Evin does not carry the appropriate traits for the ‘male gaze’, which is not just being pretty or attractive, but is related to racial origin, to being ‘white’ or otherwise. Evin’s case shows the trajectory of ‘racism’ and ‘sexism’ in the film and media industry in Sweden. This trajectory of ‘racism’ and ‘sexism’ also enforces a set of behaviours and attitudes from Evin or, more generally, non-white women or those with Muslim background on the screen, by controlling their images. ‘Controlling images’ here dictates how a woman’s body should look on the screen, appropriate to assumed/biased cultural norms, while also creating ‘docile bodies’ ( Foucault, 1991) framed as passive entities subjected to multiple sources of intrusion, control, and discipline. As films shape cultural attitudes ( Thornham, 1999), the ‘defective femininity’ of non-white actresses like Evin reflected on the screens is related to a variety of stereotypical identity categories drawn for those with migrant history, creating an acting style for her. Creating a style for such actors, as Eckert and McConnell-Ginet (2003) note, involves “a process that connects combinations of elements of behaviour and social meaning”. Accordingly, in Evin’s case the managing film crew (producer, writer, director) not only dictates appropriate (lack of) make-up or clothes for Evin but also requires specific mimicry and gestures from her, just like other non-white or non-Swedish actresses. For instance, Evin says, actresses of Middle Eastern origin in Nordic productions are expected not to look confident, carefree, free-spirited or outgoing while playing their roles. She remembers one day she had an interview with another Kurdish scriptwriter and director, Dimen Abdulla, on a university radio station, in which they talked about sex, and the audience, other drama students and lecturers, reacted in a way that indicated they were surprised to hear two Middle Eastern women speak publicly about sex. Her response to this is “of course we talk about sex, we also have sex”. ^22^ This anecdote fits with Brune’s comprehensive report on the image of the ‘victimized woman’ in Swedish media, who is often described as a Muslim and as a person with a static ethnic and cultural identity who is assumed to lack all the characteristics of the idealized conception of Swedish woman as free, equal, and sexually emancipated ( Brune, 2002: 379–380). ^23^ This is necessary in order to be considered culturally appropriate for the region (Middle Eastern in Evin’s case) that her non-white skin is representing. So Evin’s ‘deficient’ or ‘defective’ femininity should also correspond to the cultural and patriarchal appropriateness of the ‘assumed’ gender roles in the context of her origins.

Therefore, ‘sexism’ and ‘racism’ intersect, while framing Evin as ‘undesirable’ within her ‘defective femininity’, which is also aesthetically appropriate in the framework of an essentialized image of whiteness in the Swedish/Nordic context. “Whiteness as racial categories and lived identity” ( Sunderland, 1997) also assumes and (re)produces a so-called ‘real identity’ for her as a ‘non-white’ who comes from an assumed ‘conservative’ and religious culture. So, through oppressive erasure of her ‘femininity’ and ‘sexuality’ in the roles she takes and the expectations about the nature of her performance (e.g. shy, reserved, conservative) in a Nordic context, she becomes the product of a particular cultural–political context and of the specific social dynamics within an assumed culture with which she is associated.

## From ‘defective femininity’ to ‘appropriate femininity’: mimicry or symbolic resistance?

While white feminists theorize the female image in terms of objectification, fetishization and symbolic absence, their Black counterparts describe the body as the site of ‘symbolic resistance’ in the postcolonial context ( Hammonds, 1997; Isoke, 2013; Spillers, 1987). Similarly, Evin decides to use her body as a means of ‘symbolic resistance’, destabilizing normalized racialized beauty and aesthetics by taking the role of a sexy woman, in the hope of articulating subjectivity and agency. During our meeting in February 2020, she sounded much keener on resisting desexualized roles and challenging the essentialized understanding of ‘beauty’ and expected attitudes from a non-white migrant actress. Accordingly, in 2021, she took the lead female role in *Snabba Cash* (Easy Cash), released in April 2021 - the new Swedish Netflix Original series from screenwriter Oskar Söderlund and bestselling author Jens Lapidus, who wrote the Stockholm noir trilogy of which *Snabba Cash* is the first book, adapted into three feature films.

The hugely successful and critically acclaimed *Snabba Cash* film trilogy is produced by SF Studios. Evin Ahmad, in the lead role of Leya, plays a single mother who is determined to take what she wants regardless of the danger in a male-dominated criminal world. Evin told me that Leya is her dream role, with a powerful, modern outlook, whose dark skin is not seen as a veil to cover her femininity as had been the case in previous films. ^24^ Throughout the series, in which Evin is presented ‘as subject with sexual autonomy’, she transgresses fixed boundaries for non-white actresses by carrying white actress identifiers (desirable, cis-white woman, attractive, confident, outgoing, feminine, brave). It might be thought that Evin reproduces the conventional Western body project. *Snabba Cash* is a way of entering the mainstream of everyday life. It can be argued that Evin’s character in *Snabba Cash* is constructed on another stereotype, which is that gangs and drugs are managed by migrants or members of the Black community. However, it is a sexy and dazzling lead role where her femininity and strong, smart characterization constitutes resistance, which engenders a transformative impact on the way non-white, non-native and migrant actresses are presented. This interpretation may lead to debates about ‘mimicry’ ( Bhabha, 1984) of the role of colonizer. These concepts are used in the context of colonial power and colonialist relations, but in adopting a cultural approach they can be applied to the context of the film industry in a Nordic country, with its white dominance and exclusivity, as Evin portrays an attractive and feminine cis-woman as a ‘non-white’ (colonial subject), a role usually given to ‘white’ actresses (colonizers) because the attributes of an attractive and feminine cis-womanhood are associated with whiteness. By taking the role of Leya, Evin aims to “transgress racial boundaries” ( Young, 1996) in which she had been confined by default, and all the stigmatized attributes of the characters she had played. To postcolonial and critical theorist Homi Bhabba, when the colonial subject ‘mimics’ the colonizer’s cultural habits, assumptions, institutions and values, the result is not reproduction of those traits but a ‘blurred copy’ of the colonizer. So, for Bhabba, ‘mimicry’ is not a representation of resistance although it undermines the powerful systems enacted by the colonizer or can even be seen as a subversive tool.

However, this essay argues that Evin, through the role of Leya, neither takes on white identity to escape ‘racial oppression’ ( Kennedy, 2001) nor mimics the colonizer by adopting white traits. Instead, she challenges and even attempts to change the default privileges of ‘whiteness’ that native Swedish actresses already possess without actually acting on it, because the privileges (or ‘appropriate femininity’ mentioned above) were already structured by institutional white dominance and the values whereby white bodies became “somatic norms” ( Puwar, 2004). With this role, Evin does not feel ‘out of place’ in ‘white spaces’ with her non-white body, instead she opens up a space for equality with her colleagues of Swedish origin by crossing the stigmatized racial, gender and sexual boundaries imposed on her as an actress due to her ethnicity.

## Conclusion

For more than three decades Black feminist film theorists have been debating how race, ethnicity and class intersect, and tracking modern representational embodiments of the encounter between ‘whiteness’ and its own racial meanings for Black communities in the US context. There is also a need to reassess the representation of non-western actresses, not necessarily Black, in a European and Nordic context, where it is not straightforward for an actress of Middle Eastern origin to be detached from portrayals of victimhood, shyness, obedience and restrictive traditions through the roles and characterizations imposed on them.

The increasing number of Swedish women directors, producers and screenwriters in the past decade has shown that by relevant parties, especially the Swedish Film Institute, applying a systematic approach, it is possible to improve if not completely achieve gender equality in the film industry. However, the efforts to improve gender equality do not focus on the practical or ideological implications of cinematic racial practices, especially for actresses of foreign background on screen, although notions of racial difference are routinely dramatized by filmmaking crews and expressed through film and visual media technology. Although Sweden does not have a history of racial stratification like the USA, the case of Evin Ahmad highlights how racialized standards of beauty and aesthetics based on Western norms lead to a pervasive blindness to processes of racialization on the Swedish screen. Skin functions as a boundary or border and as a mechanism for social differentiation ( Ahmed, 2000). Regardless of the fact that she was born and raised in Sweden, even though her first language is Swedish, and although she embraces Swedish culture and society with a full heart, she is still not considered Swedish, simply due to her skin colour.

Analysis of Swedish-Kurdish actress Evin Ahmad’s experiences of not being white in a white world has led me to three conclusions: (i) essentialized fixed attributes produced by white Eurocentric ideals and norms around the dominant beauty and sexualization paradigm can be imposed on actresses with a foreign background, even when the ability to obtain lead roles or achieve recognition in the industry at both national and international level is not an issue; (ii) the oppositional difference occasioned by ‘race’ or ethnic background not only leads to the reproduction of ‘controlling images’ based on historical tropes of Muslim women but also to a certain style of acting (e.g. conservative, obedient, reserved); (iii) taking the role of a character based on Western norms (e.g. desirable, cis-white woman, attractive, confident, outgoing, feminine), is not ‘mimicry’ but symbolic resistance or intervention to destabilize socio-racial orderings and erode the political and symbolic implications of white beauty. Within the framework of the naturalization of binaries and hierarchies between certain racial types in the television and film industries, it would not be surprising to find that non-white male actors would also be cast in supporting roles rather than leading ones, but one also wonders if male bodies are framed or portrayed in a form to correspond to ‘appropriate masculinity’, with actors thereby consenting to maintaining the ideology of white exclusivity.

## Ethics and consent

This project received ethical approval on 17 ^th^ July 2018 for the University of Oxford's Central University Research Ethics Committee (CUREC) for the interviews and the fieldwork (approval number: 18069). Written informed consent from Evin Ahmed was obtained and she been shown a copy of this article prior to publication.

## Data Availability

Open Science Framework: From Kurdistan to Europe https://doi.org/10.17605/OSF.IO/RBHZG ( Galip, 2023) The project contains the following underlying data: Interview with Evin Ahmad.pdf Data are available under the terms of the Creative Commons Zero “No rights reserved” data waiver (CC0 1.0 Public domain dedication).
