# Supplementary material for: Talent has no race, has no face - but it has a skin colour: The ‘appropriate femininity’ with the case of Kurdish-Swedish Actress Evin Ahmad
Source: Open Res Eur. 2023 Nov 10;3:194. [Version 1] doi: 10.12688/openreseurope.16156.1 (PMC10993007; doi:10.12688/openreseurope.16156.1)
Supplement: Peer review history

# Reviewer report: Reviewer response for version 1

**DOI:** 10.21956/openreseurope.17442.r37910

**Published:** 2024-04-03

**Authors:** Stéphane Narcis

**Affiliations:** 1 Académie de La Réunion, Réunion, France

The abstract effectively highlights the intersectional challenges faced by actresses with foreign backgrounds, particularly non-white individuals, in the Swedish film industry. It emphasises the need to examine how non-Western body images are racialised within Western beauty standards, shedding light on a neglected aspect of feminist critique in film studies. By focusing on Evin Ahmad's experiences, the article aims to illustrate the complexities surrounding race, skin colour, and body image within beauty and body culture. Incorporating feminist film critique and insights from scholars such as Homi Bhabha, Sara Ahmed, and Patricia Hill-Collins enriches the analysis, providing a comprehensive understanding of the issues. Overall, the abstract is an insightful introduction to the article's central themes and arguments, setting the stage for a nuanced exploration of race, gender, and representation in the Swedish film industry.

 The article thoroughly examines Evin Ahmad's experiences as a non-white actress in the Swedish film industry, shedding light on the intersection of race, gender, and identity in the context of film representation. The research delves into various dimensions of Evin's career, from her early roles to her recent breakthrough in the series "Snabba Cash," offering critical insights into the challenges faced by non-white actresses in predominantly white spaces.

 Strengths of the Article:

 1. Theoretical Framework: The article is grounded in a robust theoretical framework, drawing on concepts from feminist theory, postcolonial studies, and critical race theory. By integrating ideas from scholars such as Bell Hooks, Patricia Hill Collins, and Homi Bhabha, the research provides a nuanced analysis of the power dynamics at play in the film industry.

 2. Interdisciplinary Approach: The author adopts an interdisciplinary approach, incorporating perspectives from sociology, anthropology, and cultural studies. This multidimensional lens allows for a comprehensive understanding of the complex issues surrounding representation and identity in film.

 3. Empirical Evidence: The article is enriched by empirical evidence gathered through interviews with Evin Ahmad and analysis of her roles in various productions. The research offers valuable insights into the realities of non-white actresses in the Swedish film industry.

 4. Historical Context: The author contextualises Evin's experiences within broader historical narratives, such as the legacy of Orientalism and colonialism. This historical framing adds depth to the analysis and highlights the enduring impact of past injustices on contemporary representations of race and identity.

 5. Ethical Considerations: The research demonstrates a commitment to ethical standards, explicitly mentioning obtaining informed consent from Evin Ahmad and receiving ethical approval for the study. This transparency enhances the credibility of the research and underscores the importance of respecting participants' rights.

 6. Clarity and Coherence: The article is well-structured and articulated, guiding the reader through complex theoretical concepts and empirical findings with clarity. The author's writing style is engaging, making the research accessible to academic and non-academic audiences.

 7. Critical Reflection: The article is open to critical reflection on the limitations of existing discourses and the complexities of identity politics. The research encourages readers to question established norms and assumptions by interrogating concepts such as mimicry and resistance.

 Overall Assessment:

 The article contributes significantly to scholarship on race, gender, and representation in the film industry. The research's rigorous analysis and interdisciplinary approach deepen our understanding of the structural inequalities that shape cinematic narratives and offer valuable insights for future research and advocacy efforts. Its combination of theoretical rigour, empirical richness, and ethical integrity makes it a standout example of scholarly inquiry into the complexities of identity in contemporary media landscapes.

 Thanks for sharing your knowledge with the world.

Is the argument persuasive and supported by appropriate evidence?

Yes

Is the work clearly and cogently presented?

Yes

Is the topic of the essay discussed accurately in the context of the current literature?

Yes

Does the essay contribute to the cultural, historical, social understanding of the field?

Yes

Reviewer Expertise:

French / Film / Race / Sociology Studies

I confirm that I have read this submission and believe that I have an appropriate level of expertise to confirm that it is of an acceptable scientific standard.

# Reviewer report: Reviewer response for version 1

**DOI:** 10.21956/openreseurope.17442.r36232

**Published:** 2024-01-02

**Authors:** Necla Acik

**Affiliations:** 1 Law and Social Sciences, Middlesex University London, London, England, UK

This article offers a compelling analysis of the challenges encountered by Evin Ahmad, a Kurdish-Swedish actress, as she navigates the norms and representations within the film industry. The focus is particularly on her racialization and desexualisation as a ‘Middle Eastern’ actress. Grounded in feminist, critical race, and post-colonial theories, along with the concept of intersectionality of race and gender, the author delves into the processes of othering and the stereotypical representation of non-native ethnic minority actors in the predominantly white Swedish film industry.

 The author applies the Black Feminist Patricia Hill Collins’s ‘controlling images’ concept to portrayals of ethnic-minority women and actresses as passive and obedient victims, roles that pigeonhole the actor and act as barriers to professional career development. However, the author contends that this concept, developed in the US context with a focus on the White and Black binary, may not fully capture the experiences of non-Black Middle Eastern ethnic minorities in Sweden. To enhance the article, further exploration of the burgeoning literature on how ethnic minorities in Europe are racialized as ‘Muslims’ and ‘Middle Eastern,’ along with an examination of their gendered representation, would be valuable.

 Additionally, the article could benefit from a brief discussion on the racialization of ‘Middle Eastern’ in the Swedish context and clarification on why the author distinguishes the ‘Black community’ from other ethnic minorities in Sweden. The varied and sometimes contradictory terminology used to describe non-native or migrant ethnic minorities, such as ‘foreign background,’ ‘non-Swedish,’ ‘immigrants,’ ‘migrants,’ and ‘second-generation,’ introduces ambiguity. Moreover, describing Evin Ahmad as ‘non-Swedish’ appears contradictory, considering her birth in Sweden. The actor's self-identification as ‘Middle Eastern,’ Kurdish, and ‘non-white’ could be contextualized to provide clarity.

 At the core of the article lies Ahmad's defiance of stereotypes and her strategic use of roles for symbolic resistance. The narrative unfolds Ahmad's career trajectory, highlighting her deliberate choices to avoid stereotypical roles linked to honour killings and passive, obedient ethnic minority women. The analysis of her role in the Netflix series Snabba Cash exemplifies her challenge to ‘defeminized’ and ‘desexed’ images of Middle Eastern women. Her character is portrayed as strong, determined, entrepreneurial, and exhibits sexual autonomy and agency, breaking away from stereotypes.

 While Ahmad's role in the Snabba Cash disrupts stereotypical representations of non-white women in the Swedish film industry, the film also reinforces other racial representations, particularly in scenes involving gangs and drugs dominated by ethnic minorities. The author utilizes Homi Bhabha’s concept of ‘mimicry’ to analyse this paradox. Ahmad's efforts to play the role of a ‘sexy and attractive woman’ in Snabba Cash defies industry aesthetic beauty standards, distinguishing her resistance as a ‘subversive tool.’ Further development of this argument, linked with critical discussions on the British Netflix period drama's colour-blind cast, could strengthen the article. Daniella Ruiz Cantú (2022) argues that colour-blind attempts might seem progressive but, in essence, sustain white hegemony.

 In conclusion, the article makes valuable contributions to the discourse on racialized beauty and aesthetics and to scholarship on representation and agency in the Swedish and European film industry. Evin Ahmad’s case study and narratives provide a rich exploration of nuanced experiences within the intersectionality of race and gender.

Is the argument persuasive and supported by appropriate evidence?

Yes

Is the work clearly and cogently presented?

Yes

Is the topic of the essay discussed accurately in the context of the current literature?

Yes

Does the essay contribute to the cultural, historical, social understanding of the field?

Yes

Reviewer Expertise:

Kurdish Gender Studies, migration, decolonial feminism

I confirm that I have read this submission and believe that I have an appropriate level of expertise to confirm that it is of an acceptable scientific standard.

## References

[1] : Black Occularity, the White Gaze, and Color-Blindness in Shonda Rhimes’ Bridgerton dust. *https://https://scholarworks.uark.edu/cgi/viewcontent.cgi?article* .2022;
